# Case report: Pleural effusion during tyrosine-kinase inhibitor treatment in chronic myeloid leukemia: Not only a dasatinib-related adverse event

**DOI:** 10.3389/fonc.2022.1012268

**Published:** 2022-09-13

**Authors:** Raffaella Pasquale, Cristina Bucelli, Valentina Bellani, Manuela Zappa, Alessandra Iurlo, Daniele Cattaneo

**Affiliations:** ^1^ Hematology Division, Foundation IRCCS Ca’ Granda Ospedale Maggiore Policlinico, Milan, Italy; ^2^ Department of Oncology and Hemato-Oncology, University of Milan, Milan, Italy

**Keywords:** adverse event, chronic myeloid leukemia, nilotinib, pleural effusion, tyrosine-kinase inhibitor

## Abstract

The spectrum of TKI-related adverse events (AEs) is variable. Pleural effusion (PE) is a frequent AE attributable to dasatinib treatment, while it is only rarely associated with nilotinib. The pathogenetic mechanism leading to PE during nilotinib therapy is still unknown and its management has not yet been defined. To the best of our knowledge, only a limited number of similar case reports have already been reported in the literature so far. Here, we describe the case of a 41-year-old CML patient who developed PE during first-line nilotinib, successfully treated with steroids and nilotinib permanent discontinuation. We highlight the differences among our patient and the others, proposing therapeutic strategies to solve this rare but still possible AE, of which physicians should be aware.

## Introduction

*BCR::ABL1*-positive chronic myeloid leukemia (CML) is a myeloproliferative neoplasm with an incidence of 1-2 cases per 100.000 adults, which represents approximately 15% of newly diagnosed cases of leukemia in adults. This disease is characterized by a single reciprocal translocation between chromosomes 9 and 22, resulting in the formation of the Philadelphia (Ph) chromosome. *BCR::ABL1* fusion gene encodes a p210 protein (BCR::ABL1) with deregulated tyrosine kinase activity. Knowledge of this translocation was the basis for the development of drugs known as small molecule tyrosine-kinase inhibitors (TKIs) (1).

Currently, five TKIs are approved for CML treatment: original/generic imatinib, nilotinib, dasatinib, and bosutinib are recommended for both first and second or later lines, and ponatinib for second or subsequent lines, representing at the moment the only TKI that can be effectively used also in the case of the T315I point mutation. Each TKI has a distinct toxicity profile with most adverse effects (AEs) expressing ‘off-target’ toxicity of TKIs such as in the case of pleural effusion (PE) (**Table 1**) (2). This AE is reported only rarely during treatment with imatinib (1-2%) or bosutinib: considering in particular the latter TKI, PE has been reported in both the first and second or subsequent lines of treatment, with an incidence rate which varies from 1.9% to 6.1% (3–5). On the contrary, PE is a typical dasatinib-related AE with a higher incidence in patients showing baseline risk factors such as older age, history of pulmonary and/or hearth diseases, uncontrolled hypertension, hypercholesterolemia and/or autoimmune disorders (2, 6–9). In the DASISION trial, the incidence of PE at 5 years of follow-up was 28% in the dasatinib arm compared to 1% in the imatinib arm (10). A similar incidence of PE during dasatinib treatment was reported in real-life experiences, with a recurrence rate of 59.4% (11). In the ELN recommendations for the management of TKI-related AEs, recurrence of PE occurs in approximately 70% of the cases, thus representing the leading cause of dasatinib discontinuation (12).

**Table 1:** Adverse events during TKI therapy.

	IMATINIB	DASATINIB	NILOTINIB	BOSUTINIB	PONATINIB
Fluid retention	+++	–	–	+	–
Muscle cramps	+++	–	–	–	–
Fatigue	++	++	++	++	++
Rash	++	–	+++	+/-	+++
Nausea	++	+/-	+	+++	++
Diarrhea	++	–	–	+++	–
Increased pancreatic enzymes	–	–	++	+	+++
Hypertension	–	++	++	–	+++
Pleural effusion	–	+++	–	+	–
Arterial occlusive events	–	–	++	–	+++

+ means low frequent, ++ means intermediate frequent, +++ means high frequent, - means infrequent.

As for nilotinib, it has a peculiar cardiovascular (CV) toxicity reported in 20% of patients, contraindicating its use in case of previous CV diseases, while other common side effects may include hypercholesterolemia and hyperglycemia (13). In the 10-year ENESTnd analysis, approximately 40% of nilotinib-treated patients experienced vascular events (14). In contrast, PE is rare during nilotinib: in the 6-month follow-up of an open-label Phase II clinical trial, only 1% of patients treated with nilotinib developed PE and all cases were Grade 1–2 in severity (15).

## Case description

**Figure 1:**
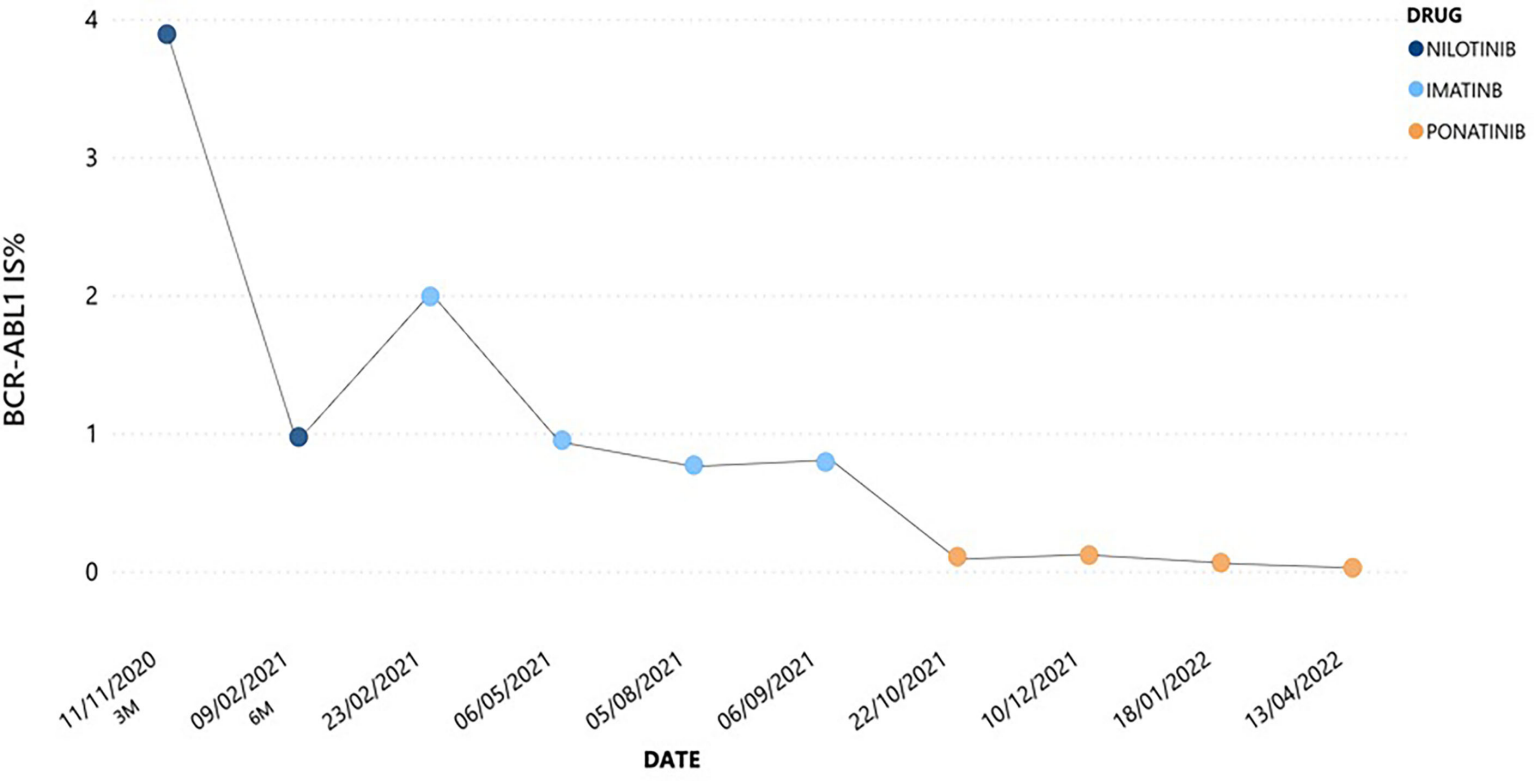
*BCR::ABL1* transcript levels during the treatment with each TKIs.

Here, we describe the case of a 41-year-old man with a history of esophageal achalasia affected by chronic phase (CP)-CML, high risk according to Sokal and low risk according to ELTS score. In July 2020 he was admitted to our hospital because of pain in left hypochondrium. Blood exams showed extreme leukocytosis [289.850/mmc with 5% of peripheral blood (PB) blasts] and thrombocytosis (903.000/mmc), associated with mild anemia and moderate splenomegaly (bipolar diameter of 17.5 cm at ultrasound examination). Given the diagnostic suspicion of a myeloid neoplasm, we searched for the *BCR::ABL1* p210 fusion transcript and the *JAK2*V617F mutation. The latter was not detected; on the contrary, a diagnosis of CML was made by means of qualitative PCR, which demonstrated the presence of a typical e14a2 (b3a2) *BCR::ABL1* p210 configuration. Bone marrow morphological analysis showed a CP-CML pattern, and cytogenetic studies revealed a 46,XY karyotype with the t(9;22)(q34;q11.2)[20/20], and no additional chromosomal abnormality. After previous debulking therapy with hydroxyurea, considering his young age, absence of any significant comorbidities and CML risk scores, first-line therapy with nilotinib 600 mg BID was started. After 3 months, the patient achieved an optimal response as defined in 2020 ELN recommendations (16), with a *BCR::ABL1* transcript level of 3.89% according to the International Scale (IS). However, at 6 months the patient complained about weight gain of 7 Kg and dyspnea on exertion, with no significant peripheral edema. An abdomen ultrasound showed neither hepato-splenomegaly nor ascites, while extensive bilateral PE with disventilative compression phenomena was collaterally detected. Chest X-ray confirmed this finding; even though there was no evidence of pericardial effusion in echocardiography, nilotinib was prudently discontinued. In addition, to exclude a concomitant lung infection, a CT scan was performed showing Grade 2 PE according to CTCAE v.4.0, and parenchymal thickening associated with shaded areas of peribronchiolar groundglass and signs of interstitial edema. Broncholwash excluded Aspergillus, respiratory viruses including SARS-CoV2, CMV-DNA, mycobacteria, Legionella pneumophila, Mycoplasma pneumoniae, Chlamydia pneumoniae, and Pneumocystis jirovecii infections. Interestingly, flow cytometry performed on broncholwash documented lymphoid cellularity for 11% distributed as follows: 66% T CD3+, CD4/CD8 = 1.6; 32% NK CD3-/CD16+; 2% B CD19+. No reactivity was instead detected for CD117 and/or CD34. Three days later, once an infectious etiology has been ruled out, steroid treatment with prednisone 25 mg QD was started. After 10 days a new chest X-ray was performed, documenting complete resolution of lung involvement together with an initial improvement in both dyspnea and body weight. Due to the severity of nilotinib toxicity and the optimal response already achieved, the drug was definitively discontinued and imatinib was started at the dosage of 400 mg QD. Imatinib therapy was well tolerated with no significant AE, in parallel with a progressive reduction in *BCR::ABL1* transcript level up to 0.7695% IS. However, after 6 months from imatinib start molecular evaluation showed a slight increase in *BCR::ABL1* transcript level till to 0.7924% IS, thus defining a warning response according to 2020 ELN recommendations (**Figure 1**) (16). Therefore, search for *ABL1* point mutations was performed, excluding any mutation known for conferring resistance to TKIs (including imatinib). Accordingly, considering the young age of the patient and the absence of any CV risk factors (score CHART 1%), third-line therapy with ponatinib at the dosage of 30 mg QD was started from September 2021. This new treatment did not lead to any significant toxicity and allowed the patient to achieve a progressive reduction in *BCR::ABL1* transcript level, till to the obtainment of a major molecular response (MMR).

## Discussion

The most common non-hematological AEs of nilotinib are already well-known, including peripheral arterial occlusive diseases, followed by QTc interval prolongation, pancreatic enzymes, bilirubin and glucose blood levels elevation, gastrointestinal symptoms, pruritus, rash, headache, fatigue, arthralgia, nasopharyngitis, fever and night sweats; on the contrary, dasatinib has a peculiar pulmonary toxicity with a high incidence of PE estimated between 14% and 30% (17).

These AEs may be due to “off-target” effects; in particular, dasatinib-induced PE may be secondary to potent PDGFR-β inhibition in association with other possible mechanisms such as SRC inhibition (18). Indeed, it should be emphasized that PDGFR-β inhibition alone cannot cause serosal inflammation: for example, this AE is not associated with sorafenib which, however, also targets PDGFR-β.

One possible explanation is that dasatinib-related PE may be secondary to the cytotoxic T and NK cells expansion or to the action of other kinases (18). Indeed, PE is usually associated with dasatinib-induced non-malignant inflammatory lymphocytosis, which is often of NK type. The drug inhibits key kinases involved in the maturation of T and B lymphocytes, sometimes causing clonal expansion of large granular lymphocytes (LGL); the latter mainly involve NK or cytotoxic T cells, which can be detected in both PB and pleural fluid. This immunomodulatory effect, with lymphocytosis and clonal expansion of LGL, which are positively correlated with the onset of PE, has also been shown to be associated with a better response to treatment (19–21). As expected, patients with autoimmune diseases or previous immune-mediated AEs related to other TKIs are at increased risk of PE during dasatinib therapy (20, 21).

Regarding bosutinib, an orally active dual SRC and ABL1 TKI with minimal activity against PDGFR or c-KIT (22, 23), it has been more rarely associated with PE (24), both in real-life experiences (25) and in randomized clinical trials: more specifically, the incidence rate of PE ranged from 1.9% in the phase III BELA trial (26) to 6.1% in the phase IV BYOND study (4). Also considering the most recent BFORE trial, which compared bosutinib *vs.* imatinib for patients with newly diagnosed CP-CML, PE occurred in 5.2% of bosutinib-treated subjects, with the most promising risk factors for this AE, in addition to bosutinib treatment, represented by advanced age, smoking habit, and history of pulmonary events (5). The mechanism of action for bosutinib is unclear; however, major immunological changes during treatment do not seem to be the predominant factor (27).

Although the pathogenesis of dasatinib-induced PE has already been elucidated, the etiology of this AE during treatment with nilotinib has not yet been described (16).

Unlike dasatinib, nilotinib is a weaker PDGFR inhibitor, thus leading to a PE incidence of less than 1% in this setting (2), while inhibiting DDR1 phosphorylation expressed on bronchial epithelial cells in the same way as dasatinib (21, 28).

Information on this specific topic is rather scarce, being mainly represented by case reports. In 2012 Chakraborty et al. reported the case of a 66-year-old male CML patient with progressive dyspnea due to PE 2-3 months after initiation of second-line nilotinib treatment. Once cardiac and pulmonary etiologies were excluded, nilotinib was discontinued and the patient was treated for community-acquired pneumonia with only slight improvement. Despite the low incidence of PE with nilotinib, a short course of steroid was initiated with progressive resolution (29). In 2014 Teke et al. reported a similar case of a 68-year-old male patient with CML treated with nilotinib after imatinib discontinuation due to skin rash. After 5 years from nilotinib start, he developed PE associated with a suspicious lung mass. Catheter thoracostomy was performed detecting exudative pleural fluid enriched in lymphocytes, but without malignant cells. For suspected neoplasia, the patient underwent surgery with a preliminary diagnosis of pulmonary malignancy, then total decortication was performed, but the subsequent cytological and pathological evaluation was negative. After exclusion of any pulmonary and cardiac etiology, this serious complication was attributed to nilotinib and treatment with diuretic and steroid was started. After approximately 1.5–2 months on steroids, PE almost completely resolved and nilotinib was resumed at a reduced dose of 200 mg BID, then increased to 400 mg BID (30). More recently, Satoh et al. reported the case of a 23-year-old CML patient on nilotinib who had already suffered from PE during dasatinib a few years earlier. Due to respiratory failure, he was admitted to the ICU where endotracheal intubation and left chest drainage were performed. After extubation, the patient’s condition gradually recovered and CML treatment was changed to ponatinib (31). Consequently, PE associated with nilotinib can be successfully treated in the same way as those related to dasatinib, including both steroids and diuretics, thus suggesting a similar pathogenetic mechanism, with inhibition of PDGFR among others. Despite the rarity of this AE, it should be considered once other possible cardiac or pulmonary causes have been ruled out. To support this specific etiology, Satoh et al. also looked for nilotinib in PB and pleural fluid and found it in both samples with a concentration of 927 and 2092 ng/mL, respectively, 60 hours after stopping nilotinib (31).

Consequently, as CML patients receiving TKIs can be expected to have a near-normal life expectancy and quality of life (QoL), individual characteristics of CML subjects, including comorbidities, lifestyle preferences, and TKI compliance, along with distinct ‘off-target’ TKI toxicities (which can lead to drug-related long-term morbidities) and molecular *BCR::ABL1* profile, are among the critical factors to consider when choosing the proper TKI, either as first, second or subsequent lines of therapy (2, 32–34).

All this considered, returning to our patient, it was decided not to restart nilotinib, even at half the standard dose, but to introduce imatinib for multiple reasons: firstly, unlike dasatinib and bosutinib, for which it is already known that they can be resumed at the same dose once the first episode of PE has resolved (12), no specific guidelines are now available for PE management during nilotinib. In support of this observation, Teke et al. resumed nilotinib at a reduced dose of 200 mg BID, then increased to 400 mg BID (30); on the contrary, Satoh et al. modified CML treatment in ponatinib (31). More important, given our patient’s young age and the lack of information on the exact pathogenetic mechanisms of nilotinib-related PE, nilotinib continuation could have exposed the patient to long-term AEs, negatively impacting on his QoL and, consequently, on TKI compliance and efficacy.

## Conclusions

In this case report, nilotinib was found to be the only possible cause of PE, after excluding other etiologies. However, unlike previous experiences, due to the severity of this rare AE and also considering the optimal response that the patient had already obtained, with the aim of preventing future PE recurrences, nilotinib was permanently discontinued with no new episodes during subsequent TKIs. In addition, some differences from other cases should be noted: firstly, this AE occurred during first-line therapy without concomitant medications. Overall, considering these experiences, it is not possible to hypothesize clear risk factors for nilotinib-induced PE with the sole exception of male sex. Indeed, differently from gender, older age does not seem to be prognostically relevant. The same was true for comorbidities: the first patient reported had coronary artery disease and hypertension, among others. The remaining patients, including the one described in this case report, had no significant comorbidities. Duration of treatment also does not appear to have an impact on PE risk as patients developed this AE within 2-3 months to 5 years of starting nilotinib. An unmet clinical need may be the best management of this AE: apart from supportive care, i.e., steroids and diuretics, the real indication for switching from nilotinib to another TKI after a single episode of PE is still unclear. In our case, due to the severity of the clinical presentation of this rare AE and the progressive reduction in *BCR::ABL1* transcript level, in order to avoid new drug suspensions due to recurrences of PE, it was decided not to restart nilotinib, not even at a lower dosage, but to change the TKI by starting imatinib in the light of its safer toxicity profile. Unfortunately, due to an inadequate response to imatinib, once any known *ABL1* mutation was ruled out, given the patient’s young age and the absence of any CV risk factors, third-line therapy with ponatinib was started at the dosage of 30 mg QD, allowing the patient to quickly achieve a MMR.

## Data availability statement

The original contributions presented in the study are included in the article/supplementary material. Further inquiries can be directed to the corresponding author.

## Ethics statement

Written informed consent was obtained from the individual(s) for the publication of any potentially identifiable images or data included in this article.

## Author contributions

All authors listed have made a substantial, direct, and intellectual contribution to the work and approved it for publication.

## Funding

This study was partially funded by Italian Ministry of Health - Current research IRCCS.

## Conflict of interest

The authors declare that the research was conducted in the absence of any commercial or financial relationships that could be construed as a potential conflict of interest.

## Publisher’s note

All claims expressed in this article are solely those of the authors and do not necessarily represent those of their affiliated organizations, or those of the publisher, the editors and the reviewers. Any product that may be evaluated in this article, or claim that may be made by its manufacturer, is not guaranteed or endorsed by the publisher.

## References

[1] Jabbour E Kantarjian H. Chronic myeloid leukemia: 2022 update on diagnosis, therapy, and monitoring. Am J Hematol (2022) 97:1236–56. doi: 10.1002/ajh.26642 35751859

[2] Iurlo A Cattaneo D Bucelli C Breccia M. Dose optimization of tyrosine kinase inhibitors in chronic myeloid leukemia: A new therapeutic challenge. J Clin Med (2021) 10:515. doi: 10.3390/jcm10030515 33535564 PMC7867069

[3] Cortes JE Kim DW Kantarjian HM Brümmendorf TH Dyagil I Griskevicius L. Bosutinib versus imatinib in newly diagnosed chronic-phase chronic myeloid leukemia: results from the BELA trial. J Clin Oncol (2012) 30:3486–92. doi: 10.1200/JCO.2011.38.7522 PMC4979199 22949154

[4] Hochhaus A Gambacorti-Passerini C Abboud C Gjertsen BT Brümmendorf TH Smith BD. Bosutinib for pretreated patients with chronic phase chronic myeloid leukemia: primary results of the phase 4 BYOND study. Leukemia (2020) 34:2125–37. doi: 10.1038/s41375-020-0915-9 PMC7387243 32572189

[5] Brümmendorf TH Cortes JE Milojkovic D Gambacorti-Passerini C Clark RE le Coutre P. Bosutinib versus imatinib for newly diagnosed chronic phase chronic myeloid leukemia: final results from the BFORE trial. Leukemia (2022) 36:1825–33. doi: 10.1038/s41375-022-01589-y PMC9252917 35643868

[6] Miura M. Therapeutic drug monitoring of imatinib, nilotinib, and dasatinib for patients with chronic myeloid leukemia. Biol Pharm Bull (2015) 38:645–54. doi: 10.1248/bpb.b15-00103 25947908

[7] Suh KJ Lee JY Shin DY Koh Y Bang SM Yoon SS. Analysis of adverse events associated with dasatinib and nilotinib treatments in chronic-phase chronic myeloid leukemia patients outside clinical trials. Int J Hematol (2017) 106:229–39. doi: 10.1007/s12185-017-2225-1 28378056

[8] Masiello D Gorospe G Yang AS. The occurrence and management of fluid retention associated with TKI therapy in CML, with a focus on dasatinib. J Hematol Oncol (2009) 2:46. doi: 10.1186/1756-8722-2-46 19909541 PMC2785832

[9] Cortes JE Jimenez CA Mauro MJ Geyer A Pinilla-Ibarz J Smith BD. Pleural effusion in dasatinib-treated patients with chronic myeloid leukemia in chronic phase: Identification and management. Clin Lymphoma. Myeloma Leuk (2017) 17:78–82. doi: 10.1016/j.clml.2016.09.012 28082112

[10] Cortes JE Saglio G Kantarjian HM Baccarani M Mayer J Boqué C. Final 5-year study results of DASISION: The dasatinib versus imatinib study in treatment-naive chronic myeloid leukemia patients trial. J Clin Oncol (2016) 34:2333–40. doi: 10.1200/JCO.2015.64.8899 PMC5118045 27217448

[11] Iurlo A Galimberti S Abruzzese S Annunziata M Bonifacio M Latagliata R. Pleural effusion and molecular response in dasatinib-treated chronic myeloid leukemia patients in a real-life Italian multicenter series. Ann Hematol (2018) 97:95–100. doi: 10.1007/s00277-017-3144-1 28971265

[12] Steegmann JL Baccarani M Breccia M Casado LF García-Gutiérrez V Hochhaus A. European LeukemiaNet recommendations for the management and avoidance of adverse events of treatment in chronic myeloid leukaemia. Leukemia (2016) 30:1648–71. doi: 10.1038/leu.2016.104 PMC4991363 27121688

[13] Cortes J Mauro M Steegmann JL Saglio G Malhotra R Ukropec JA. Cardiovascular and pulmonary adverse events in patients treated with BCR-ABL inhibitors: Data from the FDA adverse event reporting system. Am J Hematol (2015) 90:E66–72. doi: 10.1002/ajh.23938 PMC11458256 25580915

[14] Kantarjian HM Hughes TP Larson RA Kim DW Issaragrisil S le Coutre P. Long-term outcomes with frontline nilotinib versus imatinib in newly diagnosed chronic myeloid leukemia in chronic phase: ENESTnd 10-year analysis. Leukemia (2021) 35:440–53. doi: 10.1038/s41375-020-01111-2 PMC7862065 33414482

[15] Kantarjian HM Giles F Gattermann N Bhalla K Alimena G Palandri F. Nilotinib (formerly AMN107), a highly selective BCR-ABL tyrosine kinase inhibitor, is effective in patients with Philadelphia chromosome–positive chronic myelogenous leukemia in chronic phase following imatinib resistance and intolerance. Blood (2007) 110:3540–6. doi: 10.1182/blood-2007-03-080689 17715389

[16] Hochhaus A Baccarani M Silver RT Schiffer C Apperley JF Cervantes F. European LeukemiaNet 2020 recommendations for treating chronic myeloid leukemia. Leukemia (2020) 34:966–84. doi: 10.1038/s41375-020-0776-2 PMC7214240 32127639

[17] Highlights of prescribing information, FDA . Available at: https://www.accessdata.fda.gov/drugsatfda_docs/label/2018/022068s029lbl.pdf (Accessed 17 Mar 2021).

[18] Quintas-Cardama A Kantarjian H O’brien S Borthakur G Bruzzi J Munden R. Pleural effusion in patients with chronic myelogenous leukemia treated with dasatinib after imatinib failure. J Clin Oncol (2007) 25:3908–14. doi: 10.1200/JCO.2007.12.0329 17761974

[19] de Lavallade H Punnialingam S Milojkovic D Bua M Khorashad JS Gabriel IH. Pleural effusions in patients with chronic myeloid leukaemia treated with dasatinib may have an immune-mediated pathogenesis. Br J Haematol (2008) 141:745–7. doi: 10.1111/j.1365-2141.2008.07108.x 18331365

[20] Bergeron A Rea D Levy V Picard C Meignin V Tamburini J. Lung abnormalities after dasatinib treatment for chronic myeloid leukemia. Am J Respir Crit Care Med (2007) 176:814–8. doi: 10.1164/rccm.200705-715CR 17600277

[21] Kelly K Swords R Mahalingam D Padmanabhan S Giles FJ. Serosal inflammation (pleural and pericardial effusions) related to tyrosine kinase inhibitors. Targeting Oncol (2009) 4:99–105. doi: 10.1007/s11523-009-0110-4 19381453

[22] Puttini M Coluccia AM Boschelli F Cleris L Marchesi E Donella-Deana A. *In vitro* and *in vivo* activity of SKI-606, a novel src-abl inhibitor, against imatinib-resistant bcr-Abl1 neoplastic cells. Cancer Res (2006) 66:11314–22. doi: 10.1158/0008-5472.CAN-06-1199 17114238

[23] Remsing Rix LL Rix U Colinge J Hantschel O Bennett KL Stranzl T. Global target profile of the kinase inhibitor bosutinib in primary chronic myeloid leukemia cells. Leukemia (2009) 23:477–85. doi: 10.1038/leu.2008.334 19039322

[24] Cortes JE Kantarjian HM Mauro MJ An F Nick S Leip E. Long-term cardiac, vascular, hypertension, and effusion safety of bosutinib in patients with Philadelphia chromosome-positive leukemia resistant or intolerant to prior therapy. Eur J Haematol (2021) 106:808–20. doi: 10.1111/ejh.13608 33638218

[25] Tiribelli M Abruzzese E Capodanno I Sorà F Trabacchi E Iurlo A. Efficacy and safety of bosutinib in chronic phase CML patients developing pleural effusion under dasatinib therapy. Ann Hematol (2019) 98:2609–11. doi: 10.1007/s00277-019-03802-y 31529281

[26] Gambacorti-Passerini C Cortes JE Lipton JH Dmoszynska A Wong RS Rossiev V. Safety of bosutinib versus imatinib in the phase 3 BELA trial in newly diagnosed chronic phase chronic myeloid leukemia. Am J Hematol (2014) 89:947–53. doi: 10.1002/ajh.23788 PMC4305212 24944159

[27] Kreutzman A Yadav B Brummendorf TH Gjertsen BT Lee MH Janssen J. Immunological monitoring of newly diagnosed CML patients treated with bosutinib or imatinib first-line. Oncoimmunology (2019) 8:e1638210. doi: 10.1080/2162402X.2019.1638210 31428530 PMC6685516

[28] Weatherald J Bondeelle L Chaumais MC Guignabert C Savale L Jaïs X. Pulmonary complications of bcr-abl tyrosine kinase inhibitors. Eur Respir J (2020) 56:2000279. doi: 10.1183/13993003.00279-2020 32527740

[29] Chakraborty K Bossaer JB Patel R Krishnan K. Successful treatment of nilotinib-induced pleural effusion with prednisone. J Oncol Pharm Pract (2012) 19:175–7. doi: 10.1177/1078155212447530 23154573

[30] Teke H.Ü Akay OM Şahin DG Karagülle M Gündüz E Andjç N. Pleural effusion: a rare side effect of nilotinib–a case report. Case Rep Med (2014) 2014:203939. doi: 10.1155/2014/203939 25276139 PMC4174971

[31] Satoh K Morisawa S Okuyama M Nakae H. Severe pleural effusion associated with nilotinib for chronic myeloid leukemia: cross-intollerance with tyrosin kinase inhibitors. BMJ. Case Rep (2021) 14:e243671. doi: 10.1136/bcr-2021-243671 PMC8420724 34479888

[32] Padula WV Larson RA Dusetzina SB Apperley JF Hehlmann R Baccarani M. Cost-effectiveness of tyrosine kinase inhibitor treatment strategies for chronic myeloid leukemia in chronic phase after generic entry of imatinib in the united states. J Natl Cancer Inst (2016) 108:djw003. doi: 10.1093/jnci/djw003 PMC4948567 26944912

[33] Yamamoto C Nakashima H Ikeda T Kawaguchi S-I Toda Y Ito S. Analysis of the cost-effectiveness of treatment strategies for CML with incorporation of treatment discontinuation. Blood Adv (2019) 3:3266–77. doi: 10.1182/bloodadvances.2019000745 PMC6855125 31698458

[34] Ciftciler R Haznedaroglu IC. Tailored tyrosine kinase inhibitor (TKI) treatment of chronic myeloid leukemia (CML) based on current evidence. Eur Rev Med Pharmacol Sci (2021) 25:7787–98. doi: 10.26355/eurrev_202112_27625 34982440

